# Can trainability constrain physical fitness adaptations to small-sided games and high-intensity interval training in young male basketball players? a prospective cohort study

**DOI:** 10.3389/fphys.2024.1491347

**Published:** 2024-12-05

**Authors:** LiXin Wei, YaFei Zheng

**Affiliations:** ^1^ Geely University of China, Chengdu, China; ^2^ ChengDu Sports University, Chengdu, China

**Keywords:** football, athletic performance, trainability, sports training, small-sided games

## Abstract

**Introduction:**

Research on the effects of training programs involving small-sided games (SSG) versus high-intensity interval training (HIIT) has been increasing in recent years. However, there is limited understanding of how an individual's initial physical fitness level might influence the extent of adaptations achieved through these programs. This study aimed to compare the impacts of SSG and HIIT on male soccer players, while also considering the players' athleticism, categorized into lower and higher total athleticism score (TSA).

**Methods:**

A prospective cohort study was conducted over a 6-week pre-season training period, involving 43 male soccer players from regional-level teams (average age 16.5 ± 0.7 years). Players were evaluated at the start and after the 6-week period. One team incorporated SSG as a core component of their aerobic-based training, while the other team used HIIT. Evaluations included a countermovement jump (CMJ) test, a 30-meter linear sprint test, and the 30–15 intermittent fitness test (30–15 IFT). TSA was calculated to assess each player's overall athleticism level (classifying them as fit and non-fit).

**Results:**

Results revealed that non-fit players showed significantly greater CMJ improvements (mean difference: 3.0 cm; *p* < 0.005) and VIFT improvements (mean difference: 0.682 km/h; *p* = 0.002) in SSG compared to fit players. In the HIIT group, non-fit players also revealed greater improvements than fit players in CMJ (mean difference: 2.5 cm; *p* < 0.005) and peak speed in sprint (mean difference: 0.706 km/h; *p* = 0.002). No significant differences were found between groups regarding the observed improvements.

**Discussion:**

In conclusion, this study suggests that the initial level of physical fitness significantly influences the magnitude of adaptations. Specifically, players with lower fitness levels appear to benefit more from training interventions. Improvements in CMJ and aerobic capacity in SSG seem to depend on players’ fitness levels, and a similar trend is observed in HIIT for CMJ and peak speed. Individualizing training programs is recommended, with a focus on providing greater or different stimuli to more well-prepared players to ensure their continued development.

## Introduction

Improving key physical fitness attributes in soccer players, such as muscle power, aerobic capacity, and linear sprinting ability, is crucial for meeting the demands of both training sessions and competitions (Buchheit et al., 2010). Muscle power is essential for explosive actions like shooting and jumping (Campo et al., 2009), as well as for generating the power needed for acceleration and changes in direction (Northeast et al., 2019). Additionally, maintaining high velocity and linear sprinting speed has become increasingly important in soccer matches due to the growing frequency of such movements (Reynolds et al., 2021). This enables players to holder rapid defensive or offensive transitions and overtake opponents in duels (Caldbeck and Dos’Santos, 2022). Lastly, a robust aerobic capacity supports sustained physical performance by enhancing endurance, allowing players to maintain high-intensity efforts throughout the match (Radziminski et al., 2020). Therefore, implementing effective training methods to improve these physical attributes can lead to better overall performance and increased match intensity.

In soccer, both small-sided games (SSGs) and running-based high-intensity interval training (HIIT) have been shown to effectively enhance aerobic capacity (Moran et al., 2019; Clemente et al., 2021c). SSGs also support improvements in muscle power and linear sprinting ability, though results in these areas are more diverse (Clemente et al., 2021b). SSGs replicate game dynamics by reducing the size of the pitch and the number of players, while adjusting rules to promote sport-specific adaptations (Hill-Haas et al., 2011; Bujalance-Moreno et al., 2019). This approach helps players engage in conditioning training while maintaining technical skills and tactical awareness (Ometto et al., 2018). Research indicates that SSGs are as effective as HIIT in significantly improving aerobic capacity (Clemente et al., 2023), though their impact on muscle power and sprint performance is less consistent (Faude et al., 2014; Los Arcos et al., 2015). In contrast, running-based HIIT consistently enhances aerobic capacity (Clemente et al., 2021c) and, in some forms, such as sprint interval training, also improves linear sprint performance (Hang et al., 2024).

A notable methodological gap in the current literature is the lack of consideration for players’ initial fitness levels when analyzing adaptation outcomes (Clemente et al., 2021a). Additionally, there is limited understanding of how physical fitness levels influence the effectiveness of these training methods. For instance, the impact of SSGs may vary based on whether players have lower or higher fitness levels, with potential challenges in adapting the training to individual needs. This variability in stimulus and the complexity of individualizing SSGs (Clemente, 2020) raise questions about whether the effectiveness of these drills is constrained by players’ fitness levels, especially for those who are already highly fit.

Given the limited research on how baseline physical fitness levels may influence the magnitude of adaptations when comparing SSGs and HIIT, further studies are needed to understand the impact of trainability on key fitness parameters such as aerobic capacity, muscle power, and linear sprint performance. Investigating this issue not only offers an innovative approach and contributes to the body of knowledge, but it also has practical implications for coaches. Such research could help coaches select the most effective training methods for individual players, thereby optimizing the training stimulus and improving overall performance. Based on this, this study purposed to compare the impacts of SSG and HIIT on male soccer players, while also considering the players’ athleticism, categorized into lower and higher total athleticism scores (TSA).

## Methods

We have reported this article in accordance with the Strengthening the Reporting of Observational Studies in Epidemiology (STROBE) guidelines for cohort studies.

### Participants

Using convenience sampling, the following criteria were established to make players from both teams eligible for this study: (i) aged 16–18 years old (the latest stage of specialization); (ii) male; (iii) with more than 3 years of soccer experience; (iv) not injured during the observational period (6 weeks) or in the month prior to the start of the pre-season; (v) not participating in additional training sessions beyond those prescribed within the context of their soccer training; and (vi) being outfield players (i.e., excluding goalkeepers).

Following the initial assessment, each athlete’s better CMJ, 30-m linear sprint and VIFT values were recorded. To standardize the scores for each measure, z-scores were computed based on the mean and standard deviation from the 43 players. The TSA for each athlete was derived from the sum of the z-scores across these measures (Turner et al., 2019). Players were categorized into two groups based on their TSA sum of z-scores: (i) those with a lower TSA if the z-score (non-fit) was below the median (−0.2), and (ii) those with a higher TSA (fit) if the z-score was above the median (−0.2). Furthermore, for data analysis, the difference between post-preseason and pre-preseason measurements was used as the final outcome for each measure, enabling comparisons between groups.

Players were recruited from two regional-level teams competing in the same age group and division. These teams were selected due to their convenience and because they adopted SSG and HIIT strategies in their training, as part of a consultancy provided by the researchers to the coaching staff. Out of the 51 players initially identified, 6 were excluded because they were goalkeepers and 2 were excluded due to injuries at the time of the first evaluation.

Before starting the study, we extended invitations to potential participants and provided them with comprehensive details about the study’s design, procedures, associated risks, and anticipated benefits. Following this, both the participants and their legal guardians completed and signed an informed consent form. This form described their right to withdraw from the study at any point without facing any repercussions. The study received ethical clearance from the Institutional Ethical Review Board at the ChengDu Sports Univ, under the reference number 102/2024. The study was conducted in accordance with the ethical principles set forth in the Declaration of Helsinki.

### Study design and setting

This study used a prospective cohort design, tracking two teams during a 6-week pre-season period. Evaluations were conducted at the beginning and end of this period (pre-post analysis). Both teams employed SSG and HIIT strategies similarly within their groups. Consequently, half of the participants in each team were exposed to each type of training. The volume of training dedicated to each approach was consistent across the groups.

**FIGURE 1:**
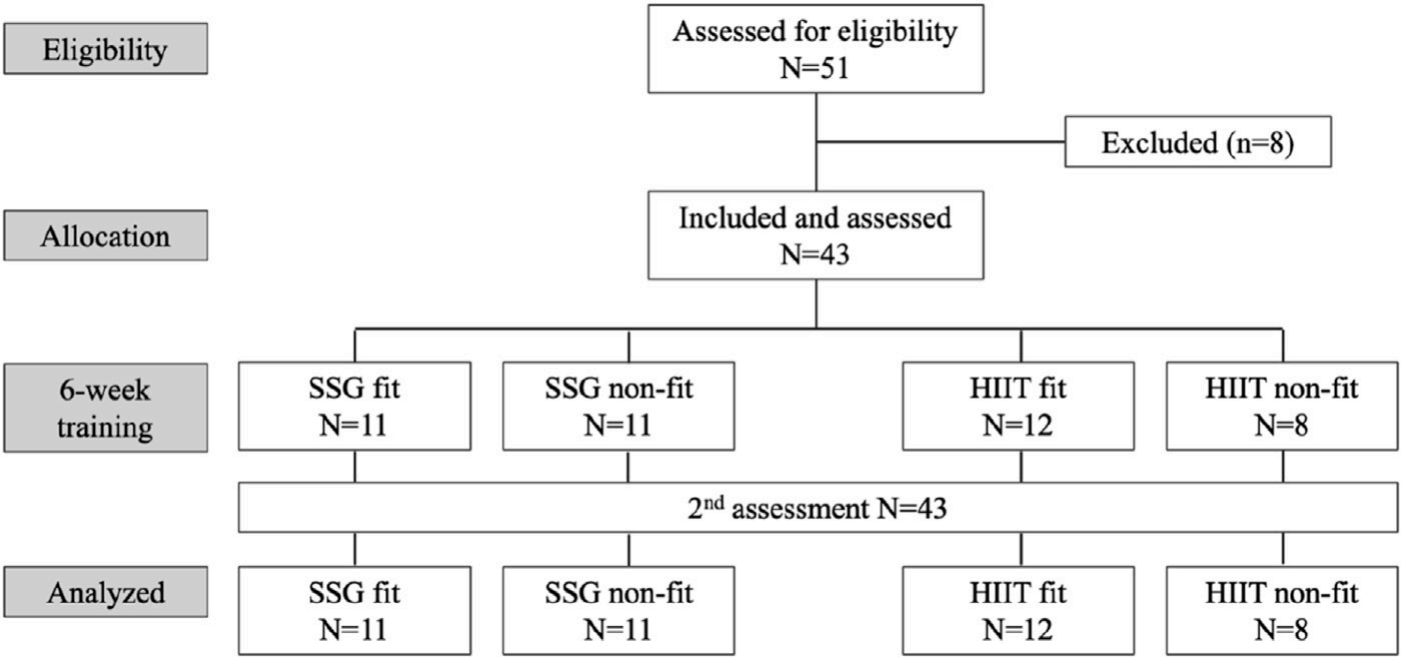
Participant Flow Chart. SSG: small-sided games; HIIT: high-intensity interval training.

The study was conducted during the pre-season period. In the first week, players were evaluated to determine their baseline physical fitness levels. Over the subsequent 6 weeks, they participated in regular training sessions (4 times a week). During these sessions, half of the participants engaged in aerobic-based training twice a week, with one group doing SSG and the other group performing HIIT. At the end of the 6-week pre-season period, a second evaluation was conducted. All players were assessed at both time points, with no dropouts recorded (Figure 1).

### SSG and HIIT training

This study investigated the potential effects of physical fitness levels on the magnitude of adaptations in players exposed to SSG and HIIT. SSG and HIIT were treated as independent variables for analysis. To ensure balance across playing positions and fitness levels, players were stratified by their roles (defenders, midfielders, and forwards). Within each positional category, a random assignment was conducted by using a coin toss, with the first toss determining the allocation for SSG and subsequent allocations alternating to ensure equal distribution. Coaches maintained these assignments consistently over the 6-week intervention period to avoid cross-training effects.

Players in the SSG group participated in two weekly sessions (separated by 48 h) of these drills, while players in the HIIT group followed a similar schedule on the same training days. Each session began with a dynamic warm-up, which included 7 min of jogging, 5 min of lower-limb stretching, and 5 min of neuromuscular exercises such as jumping and accelerations. Both SSG and HIIT sessions were part of the training dedicated to aerobic-based training.

It is important to note that the remaining sessions, focused on technical and tactical aspects, were planned and prescribed by the coaches without interference from the researchers. These sessions included one focused on strength and power and another on acceleration and linear speed. Table 1 details the aerobic training process for those exposed to SSG and HIIT.

**TABLE 1:** Description of the training process for SSG and HIIT.

	SSG	HIIT
Week 1, training 1	Reps: 6 | Time per rep: 1 min | Time between rep: 3 min	Reps: 6 | Time per rep: 1 min | Time between rep: 3 min
	Format: 2v2 | Field: 20 × 15 m | No goalkeeper | Mini-goals (2 × 1 m)	Intensity of running: 90%VIFT | Running at straight line
Week 1, training 2	Reps: 6 | Time per rep: 1 min | Time between rep: 3 min	Reps: 6 | Time per rep: 1 min | Time between rep: 3 min
	Format: 2v2 | Field: 20 × 15 m | No goalkeeper | Mini-goals (2 × 1 m)	Intensity of running: 90%VIFT | Running at straight line
Week 2, training 3	Reps: 8 | Time per rep: 1 min | Time between rep: 3 min	Reps: 8 | Time per rep: 1 min | Time between rep: 3 min
	Format: 2v2 | Field: 20 × 15 m | No goalkeeper | Mini-goals (2 × 1 m)	Intensity of running: 90%VIFT | Running at straight line
Week 2, training 4	Reps: 8 | Time per rep: 1 min | Time between rep: 3 min	Reps: 8 | Time per rep: 1 min | Time between rep: 3 min
	Format: 2v2 | Field: 20 × 15 m | No goalkeeper | Mini-goals (2 × 1 m)	Intensity of running: 90%VIFT | Running at straight line
Week 3, training 5	Reps: 3 | Time per rep: 3 min | Time between rep: 3 min	Reps: 3 | Time per rep: 3 min | Time between rep: 3 min
	Format: 4v4 | Field: 30 × 25 m | No goalkeeper | Mini-goals (2 × 1 m)	Intensity of running: 85%VIFT | Running at straight line
Week 3, training 6	Reps: 3 | Time per rep: 3 min | Time between rep: 3 min	Reps: 3 | Time per rep: 3 min | Time between rep: 3 min
	Format: 4v4 | Field: 30 × 25 m | No goalkeeper | Mini-goals (2 × 1 m)	Intensity of running: 85%VIFT | Running at straight line
Week 4, training 7	Reps: 4 | Time per rep: 3 min | Time between rep: 3 min	Reps: 4 | Time per rep: 3 min | Time between rep: 3 min
	Format: 4v4 | Field: 30 × 25 m | No goalkeeper | Mini-goals (2 × 1 m)	Intensity of running: 85%VIFT | Running at straight line
Week 4, training 8	Reps: 4 | Time per rep: 3 min | Time between rep: 3 min	Reps: 4 | Time per rep: 3 min | Time between rep: 3 min
	Format: 4v4 | Field: 30 × 25 m | No goalkeeper | Mini-goals (2 × 1 m)	Intensity of running: 85%VIFT | Running at straight line
Week 5, training 9	Reps: 8 | Time per rep: 1 min | Time between rep: 3 min	Reps: 8 | Time per rep: 1 min | Time between rep: 3 min
	Format: 2v2 | Field: 20 × 15 m | No goalkeeper | Mini-goals (2 × 1 m)	Intensity of running: 90%VIFT | Running at straight line
Week 5, training 10	Reps: 4 | Time per rep: 3 min | Time between rep: 3 min	Reps: 4 | Time per rep: 3 min | Time between rep: 3 min
	Format: 4v4 | Field: 30 × 25 m | No goalkeeper | Mini-goals (2 × 1 m)	Intensity of running: 85%VIFT | Running at straight line
Week 6, training 11	Reps: 8 | Time per rep: 1 min | Time between rep: 3 min	Reps: 8 | Time per rep: 1 min | Time between rep: 3 min
	Format: 2v2 | Field: 20 × 15 m | No goalkeeper | Mini-goals (2 × 1 m)	Intensity of running: 90%VIFT | Running at straight line
Week 6, training 12	Reps: 4 | Time per rep: 3 min | Time between rep: 3 min	Reps: 4 | Time per rep: 3 min | Time between rep: 3 min
	Format: 4v4 | Field: 30 × 25 m | No goalkeeper | Mini-goals (2 × 1 m)	Intensity of running: 85%VIFT | Running at straight line

SSG: small-sided games; HIIT: high-intensity interval training; Reps: repetitions; VIFT: final velocity at 30–15 intermittent fitness test.

### Methodological procedures

In addition to the independent variables of SSG and HIIT, we also considered the baseline physical fitness level of the participants as another independent variable in our study. Using the Total Score Average (TSA), which is derived from the average Z-scores of the testing battery, we classified participants into two groups: those above the median were categorized as “fit,” and those below the median were categorized as “non-fit.” This classification aimed to assess the impact of fitness level on the magnitude of adaptations, with the goal of determining whether trainability influences the extent of these adaptations.

The testing battery included several assessments: the countermovement jump (CMJ), measured by jump height in centimeters; a 30-meter linear sprint, measured by completion time in seconds; and the 30–15 Intermittent Fitness Test (30–15 IFT), measured by the maximum speed attained in kilometers per hour. These assessments were classified as dependent variables and were compared before and after the 6 weeks of training exposure.

### Physical fitness assessments

The evaluations were conducted under consistent conditions during both pre-season and post-pre-season analysis. On the first training session of the week, following 48 h of rest, players were assessed for muscle power, linear speed, and aerobic capacity. These assessments took place in the afternoon on synthetic turf, with temperatures averaging 24.6°C ± 1.2°C and relative humidity at 56.1% ± 2.3%. The team evaluators were the same in both moments.

The process began with the collection of demographic information (e.g., sex, birthdate), followed by anthropometric assessments (standing height and body mass). This was followed by a standardized warm-up, which included 7 min of jogging, 5 min of lower limb dynamic stretching, and 5 min of jumping and acceleration drills. After a 3-minute rest, the evaluations began, starting with the CMJ test, followed by the linear speed test, and concluding with the 30–15IFT. Players were allowed 5 min of rest between tests, and hydration was permitted during these intervals.

### Countermovement jump test

To evaluate the vertical jump height of athletes, the CMJ technique was utilized. Participants started from a standing position with their hands resting on their hips. They then performed a quick bending motion at the knees and hips, followed by an explosive upward extension of their lower body to achieve the jump. Jump height was recorded with the MyJump 2 app (version 1.0.8, Xiaomi 11i, China), known for its strong validity compared to gold-standard techniques like force plates and its established reliability (Haynes et al., 2019; Haynes et al., 2019). Each participant first completed a familiarization attempt before undertaking two jump trials, with a 1 min rest period between each. The coefficient of variation for the variability between trials was 2.8%. For subsequent data analysis, the highest jump recorded from the two attempts (measured in centimeters) was selected.

### 30-m linear sprint test

To assess linear sprint performance, a 30-meter linear sprint test was performed on synthetic turf. Participants began their sprint from a split stance, with their dominant leg positioned forward. They started their run 30 cm before the initial photocells set, ensuring they maintained the same starting position and front leg throughout the test.

A countdown in seconds signaled the start of the sprint. Three pairs of photocells were positioned at the players’ hip height: at the starting line, at the 25-meter split, and at the finish line (30 m). Each participant completed two 30-meter sprints with a 3-minute rest period between attempts. Peak speed was estimated by dividing the time taken to cover the final 5 m (between 25 and 30 m) by the duration of that split. A previous study (Zabaloy et al., 2021) have recommended this approach and found it to have nearly perfect correlations with radar gun measurements. The variability in sprint times within each participant, measured as a coefficient of variation, was 2.4%. For further analysis, the fastest of the two sprint (measured in km/h) was used.

### 30–15 intermittent fitness test

To evaluate players’ capacity for sustained intermittent exercise, the 30–15 IFT was used (Buchheit, 2008). This test involves performing a series of shuttle runs, each lasting 30 s, followed by a 15-second period of passive recovery. The pace of the runs was controlled by audio beeps, with the initial speed set at 8 km/h and increasing by 0.5 km/h every 30 s.

The test continued until the participant either could no longer keep up with the increasing pace or chose to stop due to exhaustion. The final result was recorded as the highest speed achieved during a completed 30-second interval, representing the final velocity in the 30–15 IFT (VIFT), measured in kilometers per hour (Buchheit, 2008).

### Study size

The calculation for the sample size was informed by the mean values reported in a previous comparison study of SSG versus HIIT (Nayıroğlu et al., 2022). Using G*Power (version 3.1.9.6, Heinrich-Heine-Universität Düsseldorf, Düsseldorf, Germany), which is designed for repeated measures across factors, the analysis considered four groups and two measurement points. With a significance level (alpha) set at 0.05 and a desired power of 0.85, the required sample size was determined to be 44 participants.

### Statistical procedures

In the results section, we provided descriptive statistics including means, and standard deviations. After verifying data normality and homogeneity with the Kolmogorov-Smirnov test (*p* > 0.05) and Levene’s test (*p* > 0.05), we proceeded to inferential statistical analysis. A mixed ANOVA testing interactions between time, group and fit category was conducted. A two-way ANOVA was used to compare results both groups (SSG and HIIT) and fit category (fit and non-fit). Effect sizes for the ANOVA were quantified using partial eta squared (partial $\eta^{2}$ ), with interpretations classified as follows: values greater than 0.01 indicated a small effect, values over 0.06 signified a moderate effect, and those above 0.14 represented a large effect (Richardson, 2011). Post hoc comparisons were performed using Bonferroni adjustments. Additionally, Cohen’s d effect size was used to determine pairwise comparisons, with the following classifications (Hopkins et al., 2009): 0.0–0.2 indicating a trivial effect size, 0.2–0.6 a small effect size, 0.6–1.2 a moderate effect size, 1.2–2.0 a large effect size, and values greater than 2.0 representing a very large effect size. All statistical procedures, including descriptive statistics, Kolmogorov-Smirnov and Levene’s tests, mixed repeated measures ANOVA, partial eta squared calculations, and Bonferroni tests, were executed with SPSS software (version 29.0.0, IBM SPSS Statistics, Armonk, NY: IBM Corp), and significance was set at *p* < 0.05. The graphical illustrations were conducted in JASP software (version 0.19.0, University of Amsterdam, The Netherlands).

## Results

This study included 43 male soccer players with an average age of 16.5 ± 0.7 years, a body mass of 65.1 ± 6.0 kg, and a height of 176.2 ± 5.3 cm, and an average of 5.2 ± 1.1 years of experience. Table 2 presents the descriptive statistics of the participants at both evaluation points, organized by groups and TSA classification.

**TABLE 2:** Descriptive statistics (mean ± standard deviation) of the participants at baseline and post-preseason, separated by training group (SSG vs HIIT) and TSA classification (fit vs non-fit).

	SSG fit (N = 11)	SSG non-fit (N = 11)	HIIT fit (N = 12)	HIIT non-fit (N = 8)
Baseline				
CMJ (cm)	34.8 ± 3.8	29.6 ± 3.6#	34.8 ± 3.2	29.1 ± 3.3#
30-m sprint peak speed (km/h)	28.7 ± 2.2#	28.4 ± 1.7#	28.8 ± 1.1#	27.4 ± 0.6#
VIFT (km/h)	17.1 ± 0.6#	15.9 ± 0.7#	16.5 ± 0.7#	15.8 ± 0.9#
Post pre-season				
CMJ (cm)	35.2 ± 3.2	33.0 ± 3.0#	35.3 ± 2.1	32.1 ± 2.8#
*p*-value and d-value (within-group)	*p* = 0.366; d = 0.114	*p* < 0.001; d = 1.030	*p* = 0.197; d = 0.189	*p* < 0.001; d = 0.984
30-m sprint peak speed (km/h)	29.0 ± 1.8#	28.9 ± 1.5#	29.1 ± 0.9#	28.4 ± 0.7#
*p*-value and d-value (within-group)	*p* = 0.031; d = 0.150	*p* < 0.001; d = 0.313	*p* = 0.018; d = 0.300	*p* < 0.001; d = 1.538
VIFT (km/h)	17.5 ± 0.5#	17.0 ± 0.3#	17.2 ± 0.5#	16.9 ± 0.4#
*p*-value and d-value (within-group)	*p* = 0.007; d = 0.727	*p* < 0.001; d = 2.200	*p* < 0.001; d = 1.167	*p* < 0.001; d = 1.692

CMJ, countermovement jump; VIFT, final velocity in the 30–15 intermittent fitness test, TSA, total score of athleticism. Statistically significant differences between pre- and post-season values are marked (#) at *p* < 0.05.

No significant interactions between time, group, and TSA classification were observed for CMJ (*F* = 0.368; *p* = 0.547; partial $\eta^{2}$ = 0.010), peak speed in sprint (*F* = 2.368; *p* = 0.132; partial $\eta^{2}$ = 0.059), and VIFT (*F* = 0.915; *p* = 0.345; partial $\eta^{2}$ = 0.024).

SSG non-fit participants showed a significant improvement in CMJ performance, with a mean difference of 3.4 cm (*p* < 0.001; d = 1.030, moderate effect size). Similarly, HIIT non-fit participants exhibited a significant enhancement, with a mean difference of 3.0 cm (*p* < 0.001; d = 0.984, moderate effect size). In contrast, no significant changes were observed in CMJ performance for SSG fit (*p* = 0.366; d = 0.114, trivial effect size) and HIIT fit (*p* = 0.197; d = 0.189, trivial effect size) when comparing post-intervention to pre-intervention measurements.

SSG non-fit participants showed a significant improvement in line sprint peak speed performance (*p* < 0.001; d = 0.313, small effect size), as well as fit players (*p* = 0.031; d = 0.150, trivial effect size). Similarly, HIIT non-fit participants showed significantly peak speed enhancement (*p* < 0.001; d = 1.538, large effect size), as well as fit players (*p* = 0.018; d = 0.300, small effect size). Finally, SSG non-fit participants showed a significant improvement in VIFT performance (*p* < 0.001; d = 2.200, very large effect size), as well as fit players (*p* = 0.007; d = 0.727, moderate effect size). Similarly, HIIT non-fit participants showed significantly VIFT enhancement (*p* < 0.001; d = 1.692, large effect size), as well as fit players (*p* < 0.001; d = 1.167, moderate effect size).

**FIGURE 2:**
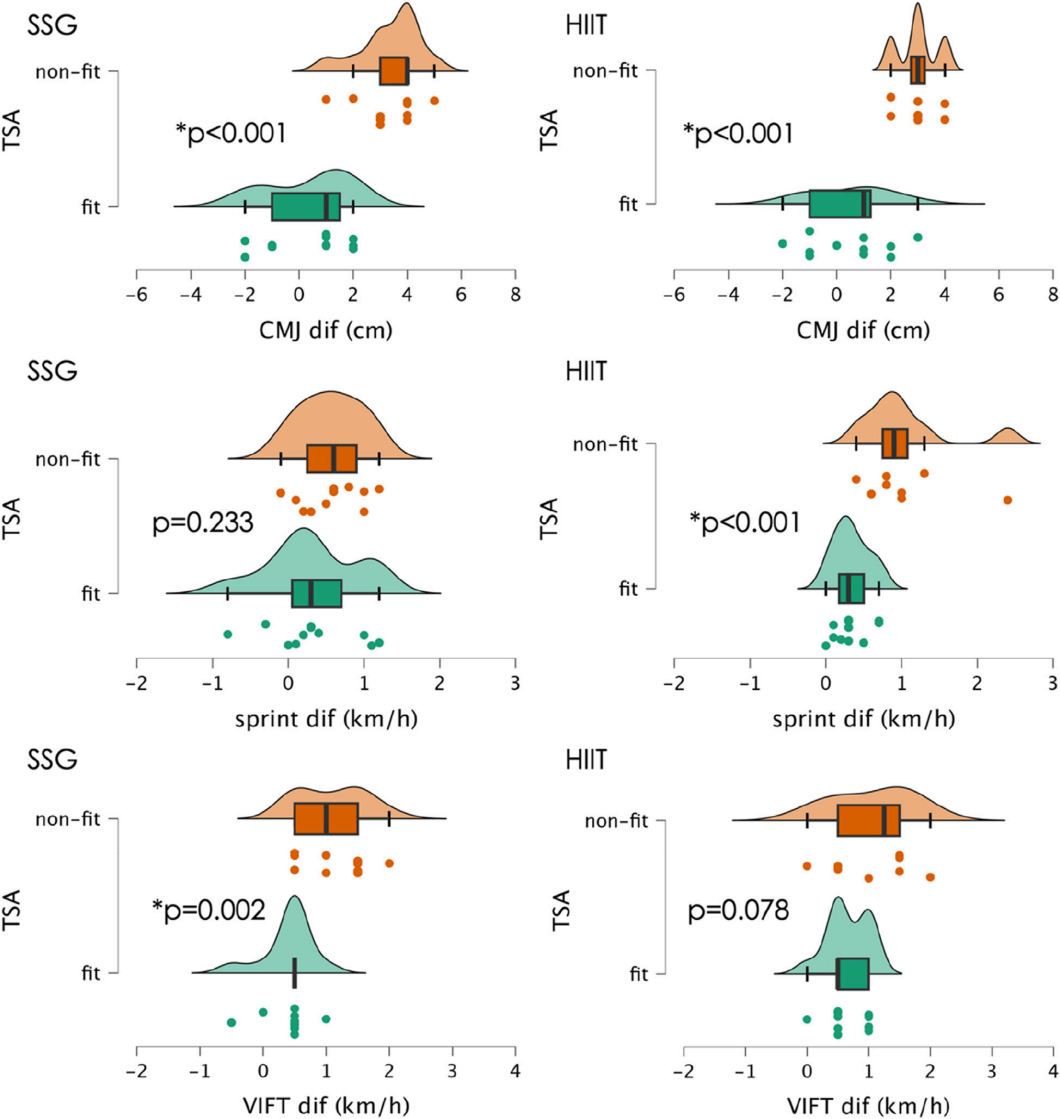
Descriptive statistics of changes in countermovement jump (CMJ), 30-meter linear sprint peak speed, and 30–15 intermittent fitness test (VIFT) between pre- and post-season assessments, categorized by training method (SSG and HIIT) and fitness group (fit vs non-fit). Error bars represent standard deviations. Asterisks (*) indicate significant differences within each group comparing fit and non-fit players (*p* < 0.05). SSG: small-sided games; HIIT: high-intensity interval training.

The Figure 2 presents the descriptive statistics of the delta variations (post-pre) for CMJ, linear sprint, and VIFT in SSG and HIIT. Non-fit players showed significantly greater CMJ improvements in SSG compared to fit players (mean difference: 3.0 cm; *p* < 0.005; d = 2.233, very large effect size). A similar trend was observed in the HIIT group, with non-fit players also demonstrating greater improvements than fit players (mean difference: 2.5 cm; *p* < 0.005; d = 2.209, very large effect size). No significant differences in CMJ improvements were observed between groups for both non-fit (*p* = 0.556; d = 0.388, small effect size) and fit players (*p* = 0.806; d = 0.089, trivial effect size).

Non-fit players showed significantly greater peak speed in sprint improvements in HIT compared to fit players (mean difference: 0.706 km/h; *p* = 0.002; d = 1.677, large effect size). However, no significant differences between fit and non-fit players were observed in SSG group (*p* = 0.233; d = 0.481, small effect size). Significant differences in peak speed in sprint improvements were observed between groups in the case of non-fit (SSG: 0.564 km/h vs HIIT: 1.037 km/h; *p* = 0.038; d = 0.923, moderate effect size), although no significant differences were found in fit players (*p* = 0.939; d = 0.045, trivial effect size).

Non-fit players showed significantly greater VIFT improvements in SSG compared to fit players (mean difference: 0.682 km/h; *p* = 0.002; d = 1.491, large effect size), although no significant differences were found in HIIT group (*p* = 0.078; d = 0.789, moderate effect size). No significant differences in VIFT improvements were observed between groups for both fit (*p* = 0.205; d = 0.735, moderate effect size) and non-fit players (*p* = 0.899; d = 0.047, trivial effect size).

## Discussion

Our study highlighted several key findings: initial fitness level and trainability significantly influence the extent of improvement. Non-fit players exhibited significantly greater improvements in CMJ, regardless of whether they participated in SSG or HIIT. They also showed enhanced peak speed in linear sprints, particularly with HIIT, and improved aerobic capacity (measured by VIFT), especially with SSG. Despite the repeated measures results indicates that both fit and non-fit players exhibit tendencies for significant within-group improvements, the study suggests that the level of initial fitness constrains the magnitude of adaptation. These findings offer valuable insights for the coaching community, emphasizing that adjustments to training stimuli may be necessary to ensure appropriate adaptations based on players’ trainability.

Our results showed that CMJ improvements were significant only in non-fit players, who experienced substantial gains from both SSG and HIIT, with no significant difference between the two training methods. It appears that the players’ initial fitness level played a more crucial role in determining the extent of improvement. Fit players, to start with, did not show any significant changes after the 6-week period, indicating that initial fitness level was a key factor in constraining the improvements. These results may help explain the often contradictory findings regarding CMJ adaptations following SSG and HIIT interventions. Some studies report significant improvements from these training approaches (Arslan et al., 2020), while others do not (Faude et al., 2014; Jastrzebski et al., 2014). Our findings suggest that the varying initial fitness levels of participants could be a key factor contributing to these differing outcomes.

The observed difference in CMJ improvements between fit and non-fit players can be explained, possibly, through the concept of a fitness ceiling (Díaz-Serradilla et al., 2023). Non-fit players exhibited significant CMJ gains from both training methods, likely due to their greater potential for adaptation in response to novel or intensified stimuli. This is consistent with the principle of progressive overload, where individuals with lower baseline fitness levels experience more substantial improvements from training as they are further from their physiological limits (Morgans et al., 2014). Conversely, fit players, who are closer to their peak physical capacity, showed insignificant changes, suggesting that their training-induced adaptations may be constrained by their existing fitness ceiling. Furthermore, the absence of a consistent and individualized neuromuscular stimulus that effectively targets neural drive, muscle force, and power may also explain the lack of improvements in this group of players (Querido and Clemente, 2020).

Our results also showed that peak sprint speed improved significantly more in non-fit players compared to fit players, but only in the HIIT group. In contrast, there were no significant differences in sprint speed between fit and non-fit players in the SSG group. Additionally, non-fit players who underwent HIIT experienced significantly greater improvements than those who participated in SSG. Our results are consistent with a study (Silva et al., 2022) that exclusively examined SSG and found no effect of baseline fitness levels on peak speed adaptations. Additionally, our study partially aligns with research suggesting that HIIT offers some advantages over SSG for enhancing sprint performance (Stojiljković et al., 2019).

Our results suggest that HIIT, even at sub-maximal speeds, although intense, as observed in our study, leads to greater adaptations, especially in individuals with lower baseline fitness levels. HIIT appears to drive more substantial neuromuscular adaptations and improvements in muscle strain and anaerobic capacity (Buchheit and Laursen, 2013), which may be more pronounced in less fit individuals who have a higher potential for improvement. In contrast, SSG, conducted in limited spaces due to the implemented formats, may not have exposed players to sufficiently high speed intensities (Castagna et al., 2017). As a result, SSG might be less effective at inducing maximal speed adaptations, regardless of baseline fitness.

The aerobic performance measured using the 30–15IFT indicated that non-fit players in the SSG group benefited significantly more than fit players. However, no significant differences between fit and non-fit players were observed in the improvements in HIIT group. No differences in the effects between the training methods was observed. Our results contrast with a previous study on SSG, which found no improvement differences between players with higher and lower TSA (Silva et al., 2022). However, our findings regarding improvements in both SSG and HIIT are consistent with recent evidence suggesting that both methods have similar beneficial effects on aerobic performance in soccer players (Clemente et al., 2023).

Non-fit players in the SSG group exhibited greater improvements compared to their fit counterparts, potentially due to the SSG’s capacity to enhance aerobic conditioning through environmental based scenarios that may be more challenging for those with lower baseline fitness. This could be attributed to increased game and tactical engagement, which may lead to more significant physiological adaptations in less fit players, thereby enhancing the training load factors that influence these adaptations (Teixeira et al., 2022; Teixeira et al., 2023). On the other hand, in those with higher fitness level, maybe the heterogeneous of the stimulus may celling the magnitude of effects (Hill-Haas et al., 2008). Conversely, the lack of different improvement between fit and non-fit players in the HIIT group points to the method’s generalized efficacy in enhancing aerobic capacity across fitness levels, individualizing the stimulus (Buchheit, 2008).

Despite the valuable insights provided by this study, several limitations should be considered. Several limitations should be considered. First, the short intervention duration (6 weeks) may not adequately reflect the long-term adaptations typically associated with SSG and HIIT. Extending the intervention could provide insights into cumulative or plateau effects that may arise over an extended training period. Additionally, conducting the study during pre-season may limit its applicability to other competitive phases, where training load and intensity differ. Furthermore, the study did not measure individual physiological responses to training stimuli, such as heart rate variability or neuromuscular adaptations, which might explain the limited response in highly trained (fit) players. In this regard, incorporating training load (Paiva et al., 2023; Teixeira et al., 2024) information and creating a modulation of adaptations based on it could be valuable in future studies. Additionally, investigating the effects of individualized training programs that consider baseline fitness levels could offer further insights into optimizing training for soccer players. F.

This study reveals the importance of adjusting training programs to players’ initial fitness levels to maximize performance gains. For coaches, the key takeaway is the necessity of individualizing training stimuli to address the specific needs of both fit and non-fit players. Non-fit players may benefit from the varied stimuli provided by both SSG and HIIT, as they have greater potential for improvement and adaptation. Conversely, fit players might require more targeted interventions to achieve further gains, given their proximity to physiological limits. Coaches may consider implementing targeted training interventions based on fitness level, with specific focus areas for fit and non-fit players. For non-fit players, SSG and HIIT can both serve as effective tools for improving explosive strength, aerobic capacity, and speed, as these athletes may respond more readily to basic conditioning stimuli. Conversely, fit players may require more tailored approaches, such as progressive overload or varied neuromuscular stimuli, to exceed existing fitness thresholds and prevent plateaus. Including regular assessments and adjusting training loads throughout the season could maximize performance gains.

## Conclusion

In conclusion, our study highlights the role of initial fitness levels and trainability in determining the effectiveness of training interventions on performance improvements. Non-fit players exhibited substantial gains across CMJ, peak sprint speed, and aerobic capacity, particularly benefiting from both SSG and HIIT. This suggests that lower baseline fitness is associated with greater potential for adaptation and improvement from diverse training stimuli. Conversely, fit players exhibited limited changes, primarily due to their proximity to physiological ceilings which constrain further gains. These findings highlight the necessity for coaches to tailor training programs based on players’ fitness levels, incorporating individualized approaches to maximize adaptations and performance outcomes.

## Data Availability

The raw data supporting the conclusions of this article will be made available by the authors, without undue reservation.
